# An Assessment of the Economic Impacts of the 2019 African Swine Fever Outbreaks in Vietnam

**DOI:** 10.3389/fvets.2021.686038

**Published:** 2021-10-25

**Authors:** Thinh Nguyen-Thi, Linh Pham-Thi-Ngoc, Que Nguyen-Ngoc, Sinh Dang-Xuan, Hu Suk Lee, Hung Nguyen-Viet, Pawin Padungtod, Thuy Nguyen-Thu, Thuy Nguyen-Thi, Thang Tran-Cong, Karl M. Rich

**Affiliations:** ^1^International Livestock Research Institute, Regional Office for East and Southeast Asia, Hanoi, Vietnam; ^2^Institute of Policy and Strategy for Agriculture and Rural Development, Hanoi, Vietnam; ^3^International Livestock Research Institute, Nairobi, Kenya; ^4^Food and Agriculture Organization of the United Nations, Hanoi, Vietnam; ^5^Department of Animal Health, Ministry of Agriculture and Rural Development, Hanoi, Vietnam; ^6^International Livestock Research Institute, Dakar, Senegal

**Keywords:** African swine fever, economic impact, multi-market model, social accounting matrix, value chain, Vietnam

## Abstract

The 2019 African swine fever (ASF) outbreaks in Vietnam imposed considerable impacts on the pig sector in Vietnam, resulting in the death or culling of nearly six million pigs, or more than 20% of the country's pig population. In order to assess the magnitude of the outbreak at sector level (both on farm and at value chain level), on livelihoods, and on the broader national economy, a comprehensive impact assessment was conducted using a mixed methods approach that integrated a value chain assessment with the use of quantitative modeling tools at sector and national levels. The results showed that the outbreak caused severe direct and indirect economic losses among farmers, particularly medium- and large-farmers whose livelihoods are largely derived from pig production. The outbreaks also affected other value chain actors due to a halving in the volume of pigs traded. At sector level, the outbreaks posed adverse impacts on the domestic supply and demand for pork, especially in the traditional sector. Meanwhile, the modern sector with higher levels of biosecurity and high technology growth was less likely to be affected and even benefited from the outbreak, which was evidenced by increased supply and income throughout the simulation period in this sector. At national level, different model simulation scenarios showed a sharp reduction in total gross domestic product (GDP) and a substantial loss of jobs. Improvements in the system of ASF compensation scheme are needed, both in terms of its administration, but also in its targeting, with greater emphasis needed on developing improved risk-sharing and funding mechanisms across national and local levels.

## Introduction

Incursions of African swine fever (ASF) can generate substantial economic losses on affected pig sectors, given its high mortality in pig populations and dislocations in pig markets (1). In East and Southeast Asia, the first ASF outbreak started in 2018 in China, home to half of the world's pig population, leading to the death and culling of 40% of its pig population (2). It has since swept across the whole region. ASF can cause up to 100% mortality in pigs and is difficult to control in the absence of an effective vaccine. As of August 2019, farmers in 51 countries have shouldered the burden of ASF, with approximately one fourth of the world's pigs killed or culled due to ASF (3).

Sharing a porous border with China, Vietnam was put on red alert as ASF had severely impacted pig farmers in China. ASF finally made its way into Vietnam with the first case reported in early February 2019. Necessary actions were implemented to prevent and control the spread of ASF in Vietnam, including a ban on the import of pigs and pork products from ASF affected countries and strict movement controls of pigs and pig products from infected provinces to the south of Vietnam. The government and relevant authorities also supported (i) early detection, culling, disinfection, and compensation, (ii) movement control, (iii) biosecurity application, (iv) risk communication and public awareness, (v) information sharing and updating, and (vi) international collaboration with donors and technical experts. Despite these strong efforts, the number of reported outbreaks and affected provinces increased rapidly. After only 5 months from the first case, ASF was found in all 62 provinces of the country, resulting in the death or culling of nearly six million pigs, or well over 20% of the country's pig population (4). More than 90% of outbreaks occurred in small- and medium-sized farms with poor biosecurity practices, which posed challenges for the prevention and control of ASF (5).

Pig production is a strategic sector of Vietnam's economy given its contribution to 60% of total livestock output (6). It is a source for the livelihoods of approximately three million households, of which 77% were smallholders (7, 8). Also, pork is the most important type of meat produced and consumed in Vietnam, representing 70% of total meat output. Therefore, the 2019 ASF outbreaks had considerable effects on the pig sector in Vietnam.

In order to assess the magnitude of economic impacts associated with the ASF outbreak at sector level (both on farm and in value chains), on livelihoods and the broader national economy, a comprehensive impact assessment was conducted. Findings from the study would allow the government and other actors to understand the scale of possible impacts and the types of investments needed to offset these negative effects. This would provide a basis for the design of necessary actions to make the response to disease and control efforts more efficient.

## Materials and Methods

A combination of qualitative and quantitative methods was used to address the multifaceted impacts of ASF in Vietnam. Details of these different methodologies are provided below.

### Assessment of ASF Impacts Along the Pig Value Chain – A Case Study

First, a case study was conducted in Duc Thang commune, Tien Lu district, Hung Yen province—the first province confirming an outbreak of ASF. Between February and June 2019, Hung Yen registered 154 outbreaks in all 10 districts and the main city, with ~135,000 pigs culled during the study period. The first ASF outbreak was detected in Tien Lu district on 12 March 2019 in one commune and then swiftly spread to all 15 communes leading to the culling of 13,920 pigs (or 24% of the district's pig population). Among the 15 communes of Tien Lu district, Duc Thang—the commune having the largest pig population—was considered to control the disease well with only 11% of its pig population being culled due to ASF.

The case study was conducted in June 2019 to assess the contextual drivers of ASF spread and control, and to determine proximate impacts among different types of value chain actors. In the case study, one focus group discussion (FGD) was organized and administered by a team of three enumerators (one facilitator, one note taker, and one board writer) to outline the pig value chain and identify potential impacts of ASF in the local context. The FGD consisted of six participants including two members of a cooperative, two independent farmers, one feed supplier, and one slaughterhouse owner who also acted as processor and retailer in the value chain. The FGD lasted 3 h. To deepen knowledge of ASF impacts on pig production and livelihoods of actors, we then carried out 19 key informant interviews (KIIs) with representatives of local authorities and the different value chain nodes. KIIs were carried out in 1 day by the same team of three members who facilitated the above FGD and each KII lasted for 30–45 min. Participants of FGD and KIIs were selected in close collaboration with the local authority and primarily based on their availability (Table 1). All participants belonged to the majority ethnic group in Vietnam (Kinh group), so the language used in FGD and KIIs was Vietnamese. Detailed interview guidelines for FGDs and KIIs were developed by staff of the International Livestock Research Institute (ILRI) and were conducted in collaboration with a team of the Vietnam National University of Agriculture (VNUA) (see Supplementary Files 1, 2). The guidelines captured information on characteristics of the actors, ways in which the ASF outbreaks affected their pig business and other farm/non-farm activities, and their reactions toward government's actions on ASF.

**Table 1:** List of participants of focus group discussion and key informant interviews.

**Respondents**	**Number of respondents**	**Note**
1. Focus group discussion (FGD)	6	
Farmers	4	Members of a cooperative (2) Independent farmers (2)
Animal feed supplier	1	
Slaughterhouse (also functioning as processor and retailer)	1	
2. Key informant interviews (KIIs)	19	
District staff	3	
Commune staff	2	
Farmers	4	Small scale (1) Medium scale (2) Large scale (1)
Broker	1	
Trader	1	
Slaughterhouse (also functioning as retailer)	2	
Slaughterhouse (also functioning as processor and retailer)	2	
Animal feed supplier	1	
Retailer	1	
Consumers	2	

Data collected from FGDs and KIIs was employed to map the value chain, identify product flows, understand the linkages among actors, and measure the impacts of ASF on the chain. Due to the small sample size, care in interpretation of the results was noted. In particular, we highlighted in section Results specific data reported by individual respondents or, where there was consensus, an aggregate value or percentage change. These data should be considered as suggestive and perceptual to contextualize the more rigorous impact assessment at sector and national levels detailed in the following two subsections. The use of small size inference to guide hypothesize building and testing on impacts was employed in the value chain space (9) and in participatory research (10).

We reported specific details of the case study in Supplementary File 3. A summary of the case study findings can be found in section Assessment of ASF Impacts Along the Pig Value Chain—key case study findings.

### Assessment of ASF Impacts at Sector Level

A variety of tools are available for measuring the impacts of animal disease at sector level (11). Multi-market partial equilibrium models, which capture the interactions of the livestock sector with related sectors (such as animal feed) are particularly useful, as they can distinguish between different production systems, while providing detailed, dynamic information on market and trade impacts of animal disease (12–14). For instance, Rich and Winter-Nelson (13) employed a multi-region, multi-species model of the livestock sector in the Southern Cone of South America to look at the impacts of different disease shocks and mitigation strategies associated with foot-and-mouth disease control.

In this part of the analysis, we deployed the Vietnam pig sector model (VPM^1^) to look at the regional and dynamic impacts of ASF at sector level and the returns to prospective intervention options based on secondary data collected from various sources (15, 16). VPM was developed in the tradition of previous spatial multimarket models of the agricultural (17) and livestock (13) sectors. VPM is a four-sector, eight-region, partial-equilibrium model that focuses primarily on the dynamics of different pig systems (traditional, commercial, and modern) and the use of maize for both human food and animal feed. Fresh pork sold in rural wet markets produced by traditional smallholder producers is categorized in the traditional sector. Fresh pork sold in urban/peri-urban wet markets produced by commercially oriented producers is categorized in the commercial sector, while processed pork sold in formal market outlets including supermarkets comprises the modern sector. The eight regions in VPM are the Northern Uplands, Red River Delta, North Central Coast, South Central Coast, Central Highlands, Southeast, Mekong River Delta, and the rest of the world. VPM simulates the evolution of the pig sector over a 13-year period starting from 2018 until 2030. Following Rich and Winter-Nelson (13), dynamics in the model over time are driven by changes in income, population, and technology, which in turn can influence the evolution of income elasticities that drive demand.

VPM was used to simulate the impact of ASF-related shocks in two scenarios: (i) a baseline scenario of income, price, and technology growth following current trends and (ii) a higher-income growth scenario. The different assumptions behind each scenario are summarized in Table 2. In all scenarios, we assumed that ASF-induced supply shocks were only applied to the traditional and commercial systems given their low levels of biosecurity. This assumption aligns with the progression of the outbreak in Vietnam in 2019, which overwhelmingly affected small- and medium-scale farms (5). Moreover, shocks to demand were differentiated by sector. We assumed a 10% rise in demand for products from the modern sector driven by consumer desires for perceived safer products. For products from the traditional and commercial sectors, we considered two levels of demand reduction, 5% and 20%, given uncertainties on how consumer demand responded to ASF outbreaks. The 5% demand shock is derived from an assumption that ASF does not significantly influence pork eating habits of Vietnamese consumers and their strong preference for fresh pork sold in wet markets. On the other hand, the 20% shock reflects consumer boycotts of pork products due to (unfounded) concerns over disease transmission from sick pigs to humans during the outbreak. We further differentiated shocks to supply and demand by region based on regional information obtained on the number of animals that were either culled or died from ASF. Finally, we imposed trade restrictions between the Northern Uplands and Red River Delta, and the Mekong River and Southeast, to simulate the effects of targeted movement restrictions that were implemented to slow the spread of ASF in the outbreak year (2019).

**Table 2:** Summary of assumptions used in the base scenario and alternative scenarios simulated in VPM.

**Scenario**	**Assumptions**
Base scenario	Per capita income growth: 5% Population growth: 1.05% Nominal exchange rate growth^a^: 1.5%; Maize technology growth: 0.5% Traditional pig technology growth: 0% Commercial pig technology growth: 1% Modern pig technology growth: 1.5% World price growth for maize: 2.08% World price growth for pork: −1.32% Income elasticity of maize: 0.4 Income elasticity of traditional pork products: 1.25 Income elasticity of commercial pork products: 1.38 Income elasticity of modern pork products: 1.51 Own price elasticity of supply for traditional pig: 0.6 Own price elasticity of supply for commercial pig: 0.65 Own price elasticity of supply for modern pig: 0.75
Higher income growth	Same as base scenario except that per capita income growth is increased to 7.5%, and Income elasticity of traditional pork products: 0.6 Income elasticity of commercial and modern pork products: 2.3

*Source: Authors' assumptions based on historic data, 2019*.

a *Nominal exchange rate growth averaged 3.15% in the period 1992–2002, 3.18% over 2002–2012, and 1.61% over 2012–2018. As there has been a downward trend in exchange rate depreciation, we chose 1.5% as our exchange rate projection for the simulation period*.

As a partial equilibrium model, the reported effects are limited to those in the pig and maize sectors, and so other impacts on agricultural and non-agricultural sectors are not reported. We address a result of the lack of data collected on these effects. However, expert consultations suggest the range of figures used in the analysis is plausible.

### Assessment of ASF Impacts at National Level

While multimarket models are useful in determining sector-level impacts, broader effects on the wider economy require different types of analytical toolkits. Social accounting matrices, or SAMs, are a type of economywide database that can be utilized to quantify the impacts of ASF on other sectors of the economy (e.g., rice, maize, vegetable, animal feed, etc.), on GDP, and employment. They expand input-output models used in economic planning by disaggregating factor and household accounts, thus allowing the analyst to conduct a distributional assessment of economic shocks on different household groups (11). In a SAM, economic activities are characterized by a set of accounts, which receive income from other activities in the economy and which purchase goods and services from other accounts. Accounts can be classified in terms of specific economic sectors as well as factors of production (labor, capital, and land) and household groups that earn and spend income from different economic sectors. SAMs follow the principle of double-entry accounting in that an account's revenues must exactly equal its expenditures (18). SAMs and input-output models have been used in a number of animal health applications to quantify macro-level impacts of animal disease incursions (9, 19–22).

We used a SAM developed by the Vietnam Central Institute for Economic Management and United Nations University-World Institute for Development Economic Research (23)^2^. The CIEM-WIDER (23) SAM is one of the most comprehensive SAMs ever constructed in a developing world setting, comprising of a set of 164 sectors, ranging from agricultural production, food processing, industrial production, and a variety of different service industries. It also distinguishes between six types of labor categories (urban and rural, each with three different levels of skills based on education level [primary, secondary, or tertiary]), agricultural and non-agricultural capital, land, and capital for livestock and fisheries. The SAM further groups households into 20 different categories, 10 each in rural and urban areas. Each rural and urban household group is further subdivided as to whether they are engaged in farming or non-agricultural activities, with each of those groups subdivided into income quintiles. While the CIEM-WIDER SAM is calibrated to 2012 data, we posit that input-output coefficients between sectors should remain robust for assessment of later periods.

The CIEM-WIDER (23) SAM was used to stimulate three scenarios that decrease the value of the supply of pigs as a result of ASF: a 10% reduction (equivalent to the volume of pigs culled as of June 2019), 25% reduction, and a worst-case 50% reduction. We extrapolated results to 2018 values by increasing the values from the 2012 SAM by 57.1%, which represents the change in GDP between 2012 and 2018. This implicitly assumes that all groups' income grows by the same amount, which will overestimate some income classifications and underestimate others. Our analysis was based on the computation of SAM multipliers and their use in scenario analysis that follow standard techniques detailed in Rich et al. (18) and Breisinger et al. (24).

In animal health applications, the focus of SAM-based analyses has typically been on GDP or national output, but SAMs can also provide useful insights on employment (25). Following the techniques described in Miller and Blair (25) and ILO (26), employment multipliers were generated to compute the number of jobs lost from different-sized outbreaks. To compute this information, we used data for 2017 from GSO on the number of jobs in sector aggregate groupings and data from the 2012 SAM on the total wage bill per sector aggregate to compute an average annual wage per sector. This was used to allocate the wage bill in the SAM by each disaggregated activity and to compute employment levels and employment/output ratios. A caveat to this approach is that it assumes discrete employment activities per sector i.e., it does not allow for employment in multiple sectors, so the values here under-estimate sectoral employment. It also does not capture informal employment; thus, some employment impacts will be under-estimated.

### Evaluation of Compensation Scheme

Finally, we analyzed the prevention and control policies by the Vietnamese government to cope with ASF in comparison with other disease outbreaks in animal, in particular highly pathogenic avian influenza (HPAI). The analysis emphasized on comparing compensation rates, eligible conditions, and financial resources for compensation based on a desk review of existing policies. We then further contextualized this analysis through our KIIs with value chain actors and an additional eight representatives of local authorities at different levels (e.g., national, provincial, district, and commune) using the KII guidelines. Our interviews helped to reveal any divergences that existed between official policy and actual implementation. The results could help policy makers understand relative performance, identify bottlenecks in their implementation, and therefore enhance improvements.

## Results

### Assessment of ASF Impacts Along the Pig Value Chain—Key Case Study Findings

ASF had a multitude of impacts on surveyed actors in the pig value chain. At farm level, we observed that larger-scale farmers were more dependent on pigs for their livelihoods than smallholder farmers. Interviewed smallholders tended to be more diversified in their sources of income, with only 20–30% of their income derived from pig production. As a result, these farmers were likely to be more insulated from outbreaks of ASF due to their reliance on other agricultural and non-agricultural activities. On the other hand, interviewed medium- and large-scale farmers were found to derive 50–100% of their income from pigs, and the ASF outbreak had much more marked impacts on livelihoods, and in particular slowed their transition toward more modernized production practices. Given the reported large reduction in pig prices by respondents (from US$1.61/kg of live weight right after the first ASF outbreak and then to US$1.39/kg at the time of the study) and an increase in production costs, particularly for biosecurity (e.g., disinfectant), the outbreak had severe consequences on the profitability of medium- and large-scale farmers. Downstream, the effects of ASF on interviewed traders, slaughterhouses, processors, retailers revealed a major shift in trading patterns, with much greater trade now occurring with large farms. These actors further experienced a sharp drop in the volume of pigs traded since the ASF outbreak that was driven by consumer fears about disease transmission from sick pigs to humans.

The study revealed significant changes in the governance of transactions along the pig chain due to ASF. Prior to the ASF outbreak, focus group discussions revealed that pig buyers could enter pig pens freely to see the pigs before deciding whether to buy or not, and payment had been made in cash on the spot. After ASF, pigs were shown to buyers through camera or apps (Zalo, Viber, etc.) rather than direct observation, and payment was transferred through bank accounts to minimize the risk of ASF transmission. Surveyed slaughterhouses noted that they became more selective in selecting pigs for slaughtering as a strategy to win customer trust and keep their reputation in the context of rising food safety concerns during ASF. Amongst surveyed farmers, enhanced collective action in the form of farmer cooperatives was effective in helping farmers cope with ASF. During the outbreak, one cooperative in the study site allocated funds to buy disinfectants and lime for members to increase disinfection around farms. Meetings were organized more regularly for cooperative farm members to update on the ASF situation, introduce effective preventive and control measures, and facilitate the supply of breeding pigs. The cooperative also proactively contacted pig traders from other provinces to purchase pigs from its members when the contracted slaughterhouses reduced capacity.

### Assessment of ASF Impacts at Sector Level

**Figure 1:**
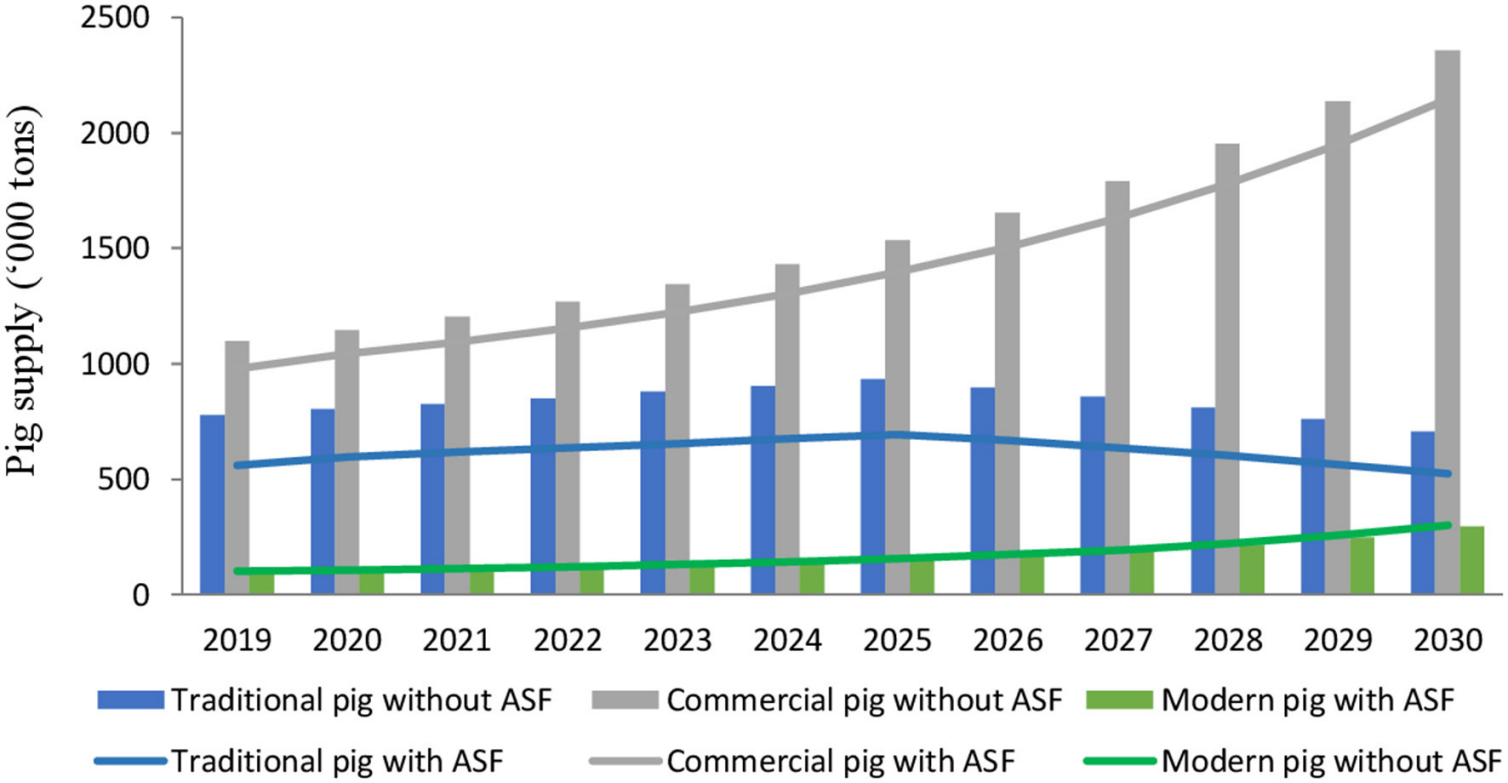
Pig supply projection with and without ASF outbreak under baseline assumptions (5% reduction in demand from traditional and commercial sectors) (Source: Model simulations). The bars are the baseline, and the lines are the level post-ASF.

Simulation results of the VPM model showed that in the baseline scenario with a 5% shock to demand for traditional and commercial pork, national pig supply falls by nearly 28% in the traditional sector, and by over 11% in the commercial sector in 2019 compared to the no-outbreak scenario (Figure 1). This is driven by sharp declines in supply, particularly in the largest production region (Red River Delta) where pork supply in the two sectors decreases by 87 and 26%, respectively. These declines persist throughout the simulation period even after the year of the outbreak (2019).

On the other hand, an ASF outbreak leads to an increase of over 5% in pig supply from the modern sector, driven by consumer preferences for perceived safer products. Supplies from the modern pig sector increase at a more modest rate of 0.5% compared to the no-outbreak scenario since 2020–2027 and start to decrease after 2028. Supply shortages trigger significant increases in the pig prices by 45% in the traditional sector, 14% in the commercial sector and 11% in the modern sector in 2019. From 2020 to 2030, similar growth rates in prices hold for traditional and commercial sector but increase at a declining rate for the modern sector.

Under this scenario, despite the negative impacts of ASF on the supply side, the total revenue of the pig sector does not fall. Rather, the losses in affected farms are offset by higher income in remaining farms due to higher prices for pork. Nationally, pig sector income in 2019 increases by just over 3% (US$89 million), with changes of nearly 4% (equivalent to US$41 million) in the traditional sector, 1.6% (US$24 million) in the commercial sector, and 17% (equivalent to US$24 million) in the modern sector compared to a no-outbreak scenario.

**Figure 2:**
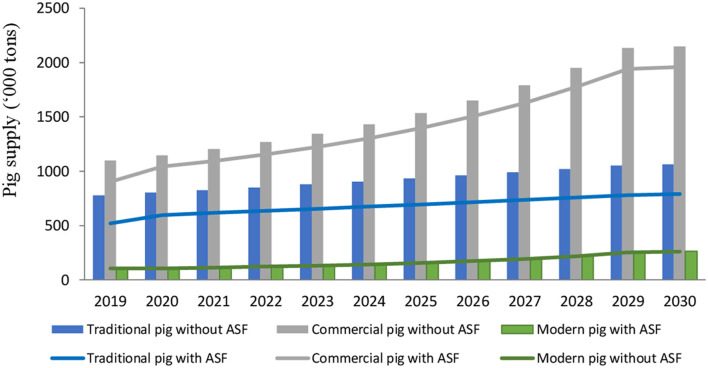
Pig supply projection with and without ASF outbreak under baseline assumptions (20% reduction in demand from traditional and commercial sectors) (Source: Model simulations). The bars are the baseline, and the lines are the level post-ASF.

If we consider a higher demand shock of 20%, we observe somewhat sharper declines in the pork supply of the traditional sector (33.2%) and the commercial sector (17.9%) compared to the previous simulation (Figure 2). Meanwhile, pig prices increase at significantly lower rates than the previous simulation (by nearly 26% in the traditional sector, but just by 0.3% in the commercial sector compared to the no-outbreak scenario). Total revenue losses under a 20% demand decline are estimated at US$420 million, led by declines in the traditional sector (nearly 16% decline, or a loss of US$172 million) and the commercial sector (18%, or a loss of US$269 million), with only the modern sector showing gains in revenue (a 14% rise, or US$20 million). These results highlight the sensitivity of our sector revenue projections to changes in demand, with more information needed to quantify how demand changed during the 2019 ASF outbreak.

Under the high-growth scenario, the impacts of an ASF outbreak on supply, demand and income are relatively similar to the previous baseline scenario but at a larger magnitude in absolute values. The Red River Delta and Southeast continue to be the most affected regions showing sharp declines in pork supply annually, especially in the traditional sector. Pig prices in all pig sectors increase at considerably larger rates (2–3 times higher than the 2019 -level) in comparison to the baseline scenario after 2026.

In both the baseline and high-growth scenarios, the share of production from the traditional sector is likely to decrease, while the commercial and modern sectors increase their shares. By 2030, ~20% of total pigs in Vietnam are produced by the traditional sector while over 70% are from the commercial and modern sector in the no-outbreak scenarios. ASF outbreaks accelerate this process, as evidenced by the shift of a 5% share in pork sales from the traditional sector to the commercial and modern sector.

We remark that our scenarios only consider ASF events affecting the smallholder sector, with the modern sector not impacted by ASF. While this aligns generally with the Vietnamese experience, this is not necessarily the case in other settings (e.g., China). Our results should be construed as a best-case scenario, as sizable outbreaks from the modern sector would imply sharper losses to the pig sector as a whole.

### Assessment of ASF Impacts at National Level

At national level, we computed the change in GDP induced by a reduction of the value of pig output from the different ASF outbreak scenarios. The SAM allows us to compute the percentage change in GDP which we applied to the 2018 value of GDP (US$245 billion) as reported by the World Bank. The best-case scenario results in a nearly 0.4% reduction in GDP, equivalent to a loss in national income of US$880 million. The worst-case scenario of a 50% loss in pig stocks is estimated to cause a decline of GDP of 1.8% and a loss in national income of US$4.4 billion (Table 3).

**Table 3:** Impacts on GDP induced by a reduction in the value of pig output caused by ASF under different scenarios.

**Scenario**	**Percentage % in GDP**	**Change in 2018 GDP (billion USD)**
10% reduction in pig output	−0.36%	−0.88
25% reduction in pig output	−0.90%	−2.20
50% reduction in pig output	−1.80%	−4.40

*Source: Computed with the 2012 Vietnam SAM, using data from World Bank to calibrate the change in GDP value (see https://data.worldbank.org/indicator/NY.GDP.MKTP.CD?locations=VN&view=chart)*.

Among the different economic sectors, feed-related sectors (prepared feed, maize, edible roots and tubers, and vegetable seeds) show sharp declines ranging from 1.76% to just over 3%. Veterinary services also fall by over 2%, while services associated with wholesaling, transport, and input provision fall more modestly but not insignificantly. The value of total economic output from the scenario is estimated to fall by 0.45% as a result of ASF. More serious outbreaks result in a larger percentage declines, with a 50% decline in the value of pigs reducing output in maize by over 13%, animal feeds by over 15%, and veterinary services by over 11% (Table 4).

**Table 4:** Sectors most negatively affected^*^ by a reduction in the value of pig output caused by ASF under different scenarios.

**Sector**	**% change in value**		
	**10% reduction in pig output**	**25% reduction in pig output**	**50% reduction in pig output**
Rice	−0.52%	−1.30%	−2.61%
Maize and other cereals	−2.63%	−6.58%	−13.16%
Edible roots and high-starch tubers	−1.76%	−4.40%	−8.80%
Oleaginous vegetable seeds	−2.00%	−5.00%	−10.00%
Other perennial crops	−0.69%	−1.72%	−3.45%
Products of pigs	−9.71%	−24.26%	−48.53%
Agricultural services	−0.59%	−1.47%	−2.94%
Vegetable and animal oils and fats	−0.78%	−1.94%	−3.88%
Prepared animal feeds	−3.01%	−7.53%	−15.07%
Pesticides and other agrochemical products	−0.72%	−1.80%	−3.59%
Basic pharmaceutical products, pharmaceutical preparations	−0.77%	−1.92%	−3.84%
Wholesale and retail trade	−0.55%	−1.37%	−2.73%
Freight rail transport services	−0.55%	−1.37%	−2.73%
Freight transport services by road, transport services via pipeline	−0.54%	−1.35%	−2.71%
Sea and coastal, inland freight water transport services	−0.55%	−1.37%	−2.73%
Freight air transport service	−0.55%	−1.37%	−2.73%
Veterinary services	−2.17%	−5.42%	−10.85%
TOTAL EFFECTS (all sectors)	−0.45%	−1.12%	−2.24%

*Source: Computed with the 2012 Vietnam SAM*.

* *Most negatively sectors are those that had a reduction in output of −0.5% or more based on the lowest output shock scenario*.

The employment effects associated with different ASF outbreak scenarios were also estimated.

Under the best-case scenario, we estimate a loss of nearly 247,000 formal sector jobs in Vietnam, of which over 86,000 occur in the pig sector, over 35,000 in wholesale and retail trade, and nearly 25,000 in the rice sector. In percentage terms, the animal feeds sectors (−3.01%) and veterinary services (−2.17%) face disproportionate losses. The worst-case outbreak of 50% of Vietnam's pigs culled would lead to over 1.2 million, or 2.3%, in job losses, with a 44% reduction, or over 431,000 jobs lost in the pig sector, nearly 176,000 jobs lost in wholesale trade and retail, and nearly 125,000 jobs lost in the rice sector (Table 5).

**Table 5:** Employment impacts of ASF in selected sectors under alternative scenarios.

	**10% reduction in pig output**		**25% reduction in pig output**		**50% reduction in pig output**	
	**% change**	**Change # jobs**	**% change**	**Change # jobs**	**% change**	**Change # jobs**
Rice	−0.48%	(24,944)	−1.20%	(62,361)	−2.39%	(124,722)
Maize and other cereals	−1.21%	(8,233)	−3.02%	(20,582)	−6.03%	(41,163)
Edible roots and high-starch tubers	−1.35%	(6,327)	−3.38%	(15,816)	−6.76%	(31,633)
Oleaginous vegetable seeds	−1.48%	(2,451)	−3.69%	(6,127)	−7.38%	(12,255)
Products of pigs	−8.80%	(86,284)	−22.01%	(215,710)	−44.01%	(431,419)
Fish products	−0.23%	(10,080)	−0.58%	(25,201)	−1.17%	(50,402)
Prepared animal feeds	−3.01%	(6,813)	−7.53%	(17,032)	−15.07%	(34,065)
Wholesale and retail trade	−0.55%	(35,186)	−1.37%	(87,966)	−2.73%	(175,931)
Veterinary services	−2.17%	(235)	−5.42%	(586)	−10.85%	(1,173)
Other agriculture	−0.32%	(23,548)	−0.81%	(58,871)	−1.61%	(117,741)
Other sectors	−0.17%	(42,664)	−0.42%	(106,659)	−0.84%	(213,318)
TOTAL	−0.46%	(246,764)	−1.15%	(616,911)	−2.30%	(1,233,822)

*Source: Computed with the 2012 Vietnam SAM*.

The distributional impacts of ASF outbreak scenarios on household groups are summarized in Table 6. In the best-case scenario, household income falls by nearly US$600 million in aggregate, with the lowest three quintiles receiving the highest losses in percentage terms, though these changes in percentage terms are only slightly larger than those faced by upper quintile groups. The rural farm sector in aggregate faces income declines of over US$338 million. In the best-case scenario, the change in income induced by an ASF ranges from −0.3 to −0.45% in rural areas, and −0.23 to −0.36% in urban areas, suggesting that outside specialized producers, pigs are a part of a broader diversification strategy with income shocks buffered to some extent from other agricultural and non-farm activities. Larger outbreaks magnify these effects, with the worst-case scenario leading to a reduction in household income by US$3 billion, and a reduction of rural farm income by US$1.7 billion.

**Table 6:** Impacts on household income groups induced by a reduction in the value of pig output caused by ASF under different scenarios.

**Household classification**	**Estimated total income in 2018 (million USD)**	**10% reduction in pig output**		**25% reduction in pig output**		**50% reduction in pig output**	
		**% change**	**Change in income (million USD)**	**% change**	**Change in income (million USD)**	**% change**	**Change in income (million USD)**
Urban farm—first quintile	552	−0.36%	(1.99)	−0.90%	(4.98)	−1.81%	(9.97)
Urban farm—second quintile	1,032	−0.36%	(3.71)	−0.90%	(9.27)	−1.80%	(18.55)
Urban farm—third quintile	2,291	−0.36%	(8.35)	−0.91%	(20.88)	−1.82%	(41.77)
Urban farm—fourth quintile	3,211	−0.34%	(10.79)	−0.84%	(26.96)	−1.68%	(53.93)
Urban farm—fifth quintile	5,077	−0.31%	(15.97)	−0.79%	(39.93)	−1.57%	(79.85)
Urban non-farm—first quintile	224	−0.28%	(0.64)	−0.71%	(1.60)	−1.42%	(3.19)
Urban non-farm—second quintile	923	−0.29%	(2.65)	−0.72%	(6.63)	−1.44%	(13.25)
Urban non-farm—third quintile	3,190	−0.26%	(8.25)	−0.65%	(20.62)	−1.29%	(41.24)
Urban non-farm—fourth quintile	10,532	−0.24%	(25.30)	−0.60%	(63.25)	−1.20%	(126.50)
Urban non-farm—fifth quintile	49,595	−0.23%	(113.31)	−0.57%	(283.28)	−1.14%	(566.55)
Rural farm—first quintile	8,298	−0.45%	(37.03)	−1.12%	(92.57)	−2.23%	(185.15)
Rural farm—second quintile	13,174	−0.43%	(57.02)	−1.08%	(142.56)	−2.16%	(285.12)
Rural farm—third quintile	16,800	−0.44%	(73.40)	−1.09%	(183.51)	−2.18%	(367.01)
Rural farm—fourth quintile	19,602	−0.42%	(82.18)	−1.05%	(205.45)	−2.10%	(410.91)
Rural farm—fifth quintile	21,935	−0.41%	(89.35)	−1.02%	(223.38)	−2.04%	(446.77)
Rural non-farm—first quintile	633	−0.37%	(2.33)	−0.92%	(5.83)	−1.84%	(11.66)
Rural non-farm—second quintile	1,926	−0.39%	(7.61)	−0.99%	(19.02)	−1.97%	(38.04)
Rural non-farm—third quintile	3,191	−0.35%	(11.24)	−0.88%	(28.10)	−1.76%	(56.21)
Rural non-farm—fourth quintile	5,204	−0.33%	(17.37)	−0.83%	(43.43)	−1.67%	(86.87)
Rural non-farm—fifth quintile	10,481	−0.30%	(31.31)	−0.75%	(78.27)	−1.49%	(156.54)
TOTAL	177,871	−0.34%	(599.81)	−0.84%	(1499.54)	−1.69%	(2999.07)

*Source: Computed from the 2012 Vietnam SAM. Note that the definition of household groups and quintiles stem from the SAM as constructed and are elaborated in the database itself, see https://www.wider.unu.edu/database/2012-social-accounting-matrix-viet-nam*.

### Evaluation of ASF Compensation Scheme

Since the first outbreak of ASF, the government issued three legal documents regulating different compensation rates for different periods in 2019. The compensation schemes were applied for two groups of beneficiaries including pig producers (i.e., households, farmers, cooperatives, etc.) and enterprises (Table 7).

**Table 7:** Compensation schemes for ASF infected stakeholders.

	**Resolution 02/2017/NÐ-CP (dated on 1 January 2017)**	**Resolution No. 16/NQ-CP (dated on 7 March 2019)**	**Decision No. 793/QÐ-TTg (dated on 27 June 2019)**
Compensation rates for pig producers	VND 38,000/kg (US$1.64) regardless of pig type	•For piglets and fatteners of all kinds: 80% of market price •For breeding pigs: 1.5–2.0 times higher than	•For piglets and fatteners of all kinds: VND 25,000/kg (US$1.09) of live pigs •For breeding pigs: VND 30.000/kg (US$1.30) of live pigs
Compensation rates for small and medium enterprises	Ineligible	•30% of producers' compensation rates •For great-grandparent and grandparent pigs: VND 500,000/head (US$21.7)	•For piglets and fatteners of all kinds: VND 8,000/kg (US$0.35) of live pigs •For breeding pigs: VND 10,000/kg (US$0.43) of live pigs •For great-grandparent and grandparent pigs: VND 500,000/head (US$21.7)
Fund allocation for compensation	•For mountainous and Central Highlands provinces, the central budget supports 80% of the support rate •For other centrally run cities and provinces that contribute *50% or more* of their revenues to the central budget, the provincial reserve funds shall be used for the support •For other centrally run cities and provinces that contribute * <50%* of total revenues to the central budget, the central budget supports 50% of the support rate •For provinces that have not yet been able to balance their budget revenues and expenditures, the central budget supports 70% of the support rate •For seriously affected provinces, if the local budgets cannot cover the costs (exceed 50% of the local reserve budget), the central budget will support the difference		

*Source: Authors' compilation from various policy documents*.

Resolution 02/2017/NÐ-CP applied for pig owners having pig herds culled due to ASF before 20 March 2019. According to this Resolution, pig owners received an average compensation rate of US$1.64/kg regardless of pig type. However, only those who had registered with the Commune People's Committee as farmers who raised livestock were eligible for the compensation. This Resolution gave little reason for pig farmers to actively report disease outbreaks and cull infected pigs for three reasons. Firstly, the compensation rate was relatively lower than the market prices prior to the first ASF detection. For instance, on 20 February 2019, the prices of live pigs were between US$2.11–2.41/kg in southern provinces, between US$1.94–2.11/kg in central provinces, and $1.98–2.24/kg in the North. Secondly, the issuance of a homogenous compensation rate (e.g., per live weight kg of pig regardless of types) could possibly lead to different application by different provinces based on what had occurred during previous HPAI outbreaks. During the HPAI outbreaks in 2004, while the government set a compensation rate of US$0.23 per head of poultry regardless type and weight, Hanoi applied US$0.23 per breeding poultry and US$0.46 per broiler while Ho Chi Minh City supported rates of US$0.70 per broiler of more than 8-week age, US$0.46 per broiler of <8-week age, US$0.23 per head of all poultry from 1-to-4 week age, and US$0.14 for all poultry <1 week age. Different compensation rates applied by provinces were considered as a major factor in inducing the movement of infected animals from one province to neighboring provinces and therefore enhancing disease spread (27). Lastly, the requirement of mandating registration for compensation eligibility was likely to be infeasible in the context of Vietnam, where the majority of pig farms were small-scale, located in residential areas, and did not have initial registration.

In order to promptly address the shortcomings above, the Vietnamese government subsequently released Resolution No. 16/NQ-CP on 7 March 2019. According to the updated scheme, different compensation rates were applied for different types of pigs and the rates aligned with market prices. Piglets and fatteners of all kinds were to be supported at the rate of 80% of the local market prices at the time and place of the outbreak, while breeding pigs received higher support (1.5–2.0 times). The condition of mandatory pig production registration with the Commune People's Committee as required prior to the outbreak was lifted as well. In addition to pig producers, the Resolution added small and medium enterprises (excluding those being subsidiaries or having dominant shared capital from large scale enterprises) as another beneficiary group of the compensation scheme. This category would receive support equivalent to 30% of the producer rates. The new compensation rates were regarded as meeting the expectation of those affected by ASF but could still cause difficulty in their implementation. Given the strong fluctuation of daily market prices, especially under the context of continuous ASF outbreaks, it was very difficult and time consuming to identify a market price base for setting up the compensation rates.

Different provinces still defined different ways to translate this regulation into practice. For instance, Hanoi city used the prices announced daily by CP company as a reference base. Every day from 8 a.m. to 10 a.m., the Department of Finance updated the CP prices on its website (https://sotaichinh.hanoi.gov.vn) and all districts and wards in Hanoi would utilize that price to define the compensation rates for households that had pigs culled on that day. Therefore, the rates were adjusted constantly aligning with the daily market fluctuation. Hung Yen province also referred to CP prices for determining the support rates, but the rates were only adjusted if the CP prices went up or down more than 20%. Consequently, since the effective date of the Resolution, Hung Yen province only adjusted their rates twice. From 20 March to 5 May 2019, their rates were fixed at US$2.07/kg for breeding pigs and US$1.39/kg for other pig types. After 6 May 2019, these rates were reset at US$1.63/kg and US$1.09/kg, respectively. Eight key informant interviews with the central and provincial authorities revealed that the constant adjustment of the compensation rates based on the market prices may lead to more complications in compensation procedures because the provincial department of finance that was responsible for fund disbursement required a detailed explanation of the reference base for compensation rate setup (e.g., what price, what date, and what time). In addition, the interviewees emphasized that the application of different compensation rates for households being infected in different periods would be possibly perceived as unfair if the information provided did not work well when the compensation was delivered to households.

Due to the significant losses caused by ASF, a further constraint was that the central and provincial budgets were not able to cover the compensation rates stated in Resolution No. 16/NQ-CP above. On 27 June 2019, the government issued Decision No. 793/QÐ-TTg adjusting the compensation rates down to US$1.09/kg for piglets and fatteners of all kinds and US$1.30/kg for breeding pigs, which were applied for pig producers. For small and medium enterprises, the corresponding rates were US$0.35/kg and US$0.43/kg, respectively. The new rates were established based on production cost rather than market prices as previous regulations and covered ~80% of total production cost.

In all of the legal documents above, the level of financial contribution from the central government and provincial authorities were clearly stated. While Hanoi and Ho Chi Minh city were able to mobilize their own budgets to cope with ASF, other provinces struggled with financial constraints. For instance, up until end-July 2019, Hanoi spent US$56.5 million for the control and prevention of ASF, including for compensation, with 100% of the budget from the city's reserve fund. The city completed compensation for almost 70% of infected households with the average time for disbursement ranging from 5 to 7 days in the early period of ASF outbreaks to around half month in the peak period. Meanwhile, Hung Yen province, with total estimated losses of US$20.4 million, could not meet the suggested risk sharing level of 50%, as its total reserve fund was only US$4.34 million. The province therefore had to rely on the central budget for doing compensation. Most recently, on 17 July 2019, the central government transferred US$55.2 million to support six provinces, of which Hung Yen received US$7.83 million. This partly explained the delay of compensation procedures and the uncertainty in the amount of time taken for farmers to receive compensation, which consequently influenced behaviors of those affected by ASF and the effectiveness of controlling disease spread. Our case study in Duc Thang commune in Hung Yen province showed that both local authorities and farmers had no clear idea regarding when the money would be approved and transferred to compensate farmers. The full compensation also might not be delivered all at once but in several stages over many months or years. For these reasons, many farmers decided to quickly sell pigs with ASF suspected symptoms instead of declaring outbreaks to the animal health authority. Two interviewed farmers in the case study confessed that they would attempt to sell their suspected pigs before ASF was confirmed, even at significantly lower prices, to recover a part of their investment rather than waiting for several years to get higher compensation.

The reliance on the central government for compensating farmers not only happened for ASF but also for other disease outbreaks, which was argued to influence the responsiveness of different provinces to outbreaks. For example, in a survey of six provinces heavily affected by HPAI outbreaks in 2004 including Ho Chi Minh city, Ha Tay (currently a part of Hanoi city), Thai Binh, Vinh Phuc, Tien Giang, and An Giang, only Ho Chi Minh city could quickly compensate infected farmers using its own budget, while other provinces were mainly dependent on central government resources. The actual percentage contributed by these provinces was far below the suggested levels of 50%, particularly Tien Giang (11%), and An Giang (8%) (27).

## Discussion

### Changes in Production and Sales Patterns Along the Value Chain

Despite various disease control efforts by the government, farmers, and several donor-supported projects, ASF cases increased throughout 2019. The outbreak caused severe direct and indirect losses among pig producers and other value chain actors, and significantly changed patterns of production, governance, and sales along the value chain.

With only 20–30% of income derived from pig production, smallholders tended to be more diversified in their sources of income and therefore were more insulated from outbreaks of ASF due to their reliance on other agricultural and non-agricultural activities. Medium- and large-scale producers, on the other hand, derive 50–100% of their income from pigs, and an ASF outbreak can both devastate livelihoods and prevent their transition toward more modernized production practices. Given a reduction in prices by nearly 50% and an increase in production costs, particularly for biosecurity (e.g., disinfectant), the outbreak has had severe consequences on such farmers.

The effects of ASF on downstream value chain actors were severe, which was evidenced a halving in the volume of pigs traded as reported by a number of interviewed traders, processors, and slaughterhouses. The outbreak also caused a major shift in trading patterns toward large farms with more secure pig supplies, and the increased use of technology rather than traditional face-to-face transaction modes to reduce the virus transmission risk. Also, consumers tended to shift their consumption behavior toward safer pork products which show clear, traceable origins and are supplied by trusted distribution channels, increase of online shopping and decrease of physical shop visits. Since the emergence of ASF outbreaks, modern retail channels (e.g., supermarkets, convenient stores, etc.) recorded a 20–30% increase in sales while traditional markets posted a 20–30% decline in sales (28).

In the short and medium-term, ASF-infected pig farms were encouraged to shift production to other species such as cattle or poultry. Provincial authorities tried to create favorable conditions for farmers to access necessary resources for such a production switch. For instance, Hung Yen province had several ongoing projects focusing on VietGAHP chicken production and beef cattle production. During this period, these projects were being given priority for pig farmers to be engaged in. Such a switch at large scale might result in a rapid increase of poultry and cattle herds, which raises concerns about the possibility of related disease outbreaks such as HPAI and foot and mouth disease in the future. However, from the management point of views, these diseases are considered as being controlled more easily than ASF due to the existence of vaccines. In addition, the government has already established an action plan to cope with these diseases. Most recently, on 16 July 2019 MARD sent an official dispatch No. 4981/BNN-TY to all provinces with regard to enhancing the implementation of national action plan on preventing and controlling HPAI during the period 2019–2025.

### Changes in Trajectories of Pig Production Systems

The ASF outbreak posed adverse impacts on the domestic pork supply and demand, especially in the traditional sector. Smallholder pig producers in the Red River Delta and Southeast suffered the highest losses driven from sharp declines in supply. Meanwhile, the modern sector with higher levels of biosecurity (and in the model not assumed to be impacted by ASF) and high technology growth was less likely to be affected and even benefits from the outbreak, which is evidenced by increased supply and income throughout the simulation period. While we would expect the gradual reduction in importance of the smallholder sector, particularly given Vietnam's livestock development strategy which promotes the development of commercial and modern farms, model results indicate that an ASF outbreak will accelerate this process.

Completely replacing small-scale farms with commercial-modern farms might not likely occur in the short term. In other words, smallholder farmers will continue to derive livelihoods from pig farming and meet a certain market. Thus, an important question is how best to effectively manage this transition in a manner that also buffers against disease shocks like ASF in the future.

The effectiveness of Duc Thang cooperative in facilitating sales and helping farmers cope with ASF provides insights on the role that collective action can play in this transition. In Vietnam, farmer groups can be organized through a very simple form of common interest groups which are self-managed by farmers that share a common interest or through more complicated form of cooperatives which are formally established under the Law on Cooperation (29). Improved coordination between various actors has been observed through the establishment of farmer groups through (i) encouraging farmers to adopt new technologies, such as VietGAHP, (ii) facilitating linkages between their members and input suppliers by signing contracts for buying animal feed, veterinary medicine and services, and credit with reliable suppliers to get better quality inputs at more favorable prices; and (iii) facilitating linkages with more stable market outlets, which creates win-win relationships, not only for farmers to stabilize their production but to also ensure a more stable source of products in both quantity and quality for buyers. The establishment of farmer groups has been strongly supported by the government and development projects of non-governmental organizations. Empirical evidence shows that the organization of farmer groups has many benefits, including better access to quality inputs and services, reduced exposure to production and market risks and reduced transaction costs (both in terms of input procurement and output marketing), and increased returns from pig production. For instance, Lapar et al. (30) indicated that members of cooperatives could obtain an increase of 16% in their profit margins per kg of live weight pig, based on a 25–30% decrease in production costs and 15–20% increase in selling prices. Pig traders could also reduce their costs of collecting and grading pigs by about 20%. Scholl et al. (29) also found that farmer group members had significantly larger pig herds than non-members (26.8 vs. 6.8); and the income of the farmer group members increased by US$827 per year compared to their counterparts.

Despite these encouraging results, the sustainability of these farmer groups after the intervention projects finish is still untested. Scholl et al. (29) showed that farmers identified external project interventions, not internal factors, as reasons for group success. For instance, subsidies from the projects in any form, either technical training or in-kind payments (pigs or monetary value of a pig, pig feed, financial incentives, etc.), were highlighted as key reasons for the successful operation of farmer groups. That explains why many farmer groups may have appeared to be successful at the time of project implementation but failed to maintain their operations once support from the project was withdrawn at the end of project implementation (29). Thus, in order to ensure the long-term development of these institutional models, factors such as member selection, management, trademark registration, strict quality control, and written contracts with regular customers should be given more attention.

### Impacts on Job Losses

The adverse impacts of the ASF outbreak were not restricted to the pig sector but also extended to other related sectors of the economy. For instance, the ASF outbreak depressed Vietnam's animal feed consumption, especially that used for pig feed. Prior to the ASF outbreak, pork production accounted for the vast majority of the total feed market of ~30 million tons. After the outbreak occurred, the feed industry experienced a 30–50% drop in sales (28). Consequently, and as revealed by the SAM, the outbreaks led to a significant loss of jobs in the pig sector and the relevant sectors, estimated as up to 247,000 jobs in the best-case scenario of 10% of Vietnam's pigs culled and 1.2 million jobs in the worst scenarios of 50% pigs culled. Social assistance schemes that target prospectively affected sectors, particularly those outside the immediate pig sector, should be considered and deployed to cushion the short-term impacts of ASF.

An additional consideration on the employment and livelihoods side is the degree to which smallholder livelihoods may be affected by ASF outbreaks. As noted earlier, smallholders are often not as negatively impacted as emerging commercial farmers, as smallholders diversify their income sources (31, 32). However, those farmers in crop-livestock systems or engaged in services that support pigs may face multifaceted negative impacts from ASF that compound income losses. Indeed, from the standpoint of household income, results from the SAM show that the poorest farm households and the poorest two quintiles of non-farm households had the largest negative effects from ASF, suggesting that while smallholders may avoid the pig-related losses associated with commercial farmers, they may still be impacted in other ways outside of direct effects to pigs.

### Effectiveness of the ASF Compensation Scheme

The implementation of the ASF compensation scheme was found to be inconsistent, with changing rules, heavy bureaucratic burdens on applicants, and significant delays in administration. A more transparent compensation system should be considered to improve confidence in public authorities and enable value chain actors to champion ASF control efforts rather than impeding it through rational self-interest. More generally, future compensation programs need to be rethought to more specifically encourage and reward good stewardship in the form of biosecurity investments. In a theoretical paper, Gramig et al. (33) note that compensation programs typically try to get producers to invest in both biosecurity and report disease, when in fact decisions to invest and report involve different types of information problems and need different incentive-compatible mechanisms to ensure compliance. They suggest a “carrot-and-stick” approach to de-link these different problems, with compensation indemnities used to induce investments in proper biosecurity, and a schedule of fines to ensure adequate disease reporting. Blanket compensation shifts the risk of disease wholly to government, and in the absence of risk classification undermines the ability of government to get farmers to self-insure through biosecurity investments (33).

Removing the requirement of having the farm registered (with the Commune People's Committee) in order to claim compensation could be a temporary solution in the current context of a vast number of unregistered smallholder farms affected by ASF. However, in the long term, reintroduction of farm registration as a compulsory criterion for compensation is recommended to enhance biosecurity on farms. This process will require a preparation period, for instance a 1-to-2-year period for transitioning, in parallel with a massive information and awareness campaign to communicate this new requirement.

The ASF outbreak in Vietnam rekindled debates on the modalities of compensation. The Vietnam experience to date show significant variation in compensation rates that could fuel movements that militate against disease control. The disconnect between central and local disbursement of compensation funds further complicates matters, with delays at regional level impeding local level efforts of control. At the same time, traditional compensation programs focus primarily on producers, yet the results from this paper show severe losses faced by various value chain actors (e.g., a reduction of 50% or more in traded volumes reported by traders and slaughterhouses), not to mention downstream effects in ancillary industries (animal health services, animal feed). As some of these actors can also serve as vectors for disease risk, providing adequate incentives for their compliance is also necessary. In the context of Rift Valley fever in Kenya, Rich and Wanyioke (9) proposed the idea of privately managed disease control funds based on a levy on sales that could be managed by producer organizations or cooperatives, as well as the possibility for government to back stand-by loans or letters of credit to deal with short-term cash flow disruptions. Indeed, one of the interviewees from a focus group discussion highlighted the role that could be played by livestock funds, for example a US$1/pig head checkoff fee that could be managed by a farmer association and used to co-insure against disease risk, support biosecurity investment, and/or improve production. This is particularly important to manage disease compensation at local level, where contingency budgets have proven inadequate at prompt disbursement. Given the various disease incursions that have faced the pig sector in the past decade, developing modalities for such funding mechanisms and their disbursement between national and local levels based on partnerships between the public and private sector should be encouraged.

## Conclusion

This study provides an important and comprehensive analysis of the impacts of the 2019 ASF outbreak in Vietnam using a mixed methods approach. The results highlighted the adverse direct and indirect impacts of ASF at different levels (i.e., at farm, sector, national level) as well as the effectiveness of the government's compensation scheme to respond and control ASF spread. Policy implications to better control and minimize ASF's adverse impacts in future include the improvement of market linkages along the pig value chain through the effective establishment and organization of farmer groups, social assistance to support those displaced by ASF, and improvements to the system of compensation with greater emphasis on developing improved risk-sharing and funding mechanisms across national and local levels.

## Data Availability Statement

The original contributions presented in the study are included in the article/Supplementary Material, further inquiries can be directed to the corresponding author/s.

## Ethics Statement

The studies involving human participants were reviewed and approved by the Institute Review Board at the Hanoi University of Public Health (No. 66/2019/YTCC-HD3). The participants provided their written informed consent to participate in this study.

## Author Contributions

ThinhN-T and KR conceived, designed the study, and took the lead in writing the manuscript. ThinhN-T, SD-X, HL, HN-V, and PP reviewed the pig value chain case study and analyzed the compensation scheme. LP-T-N, QN-N, ThinhN-T, TT-C, and KR ran the VPM model to quantify the impacts of ASF at sector level. KR used the SAM to analyze the ASF impacts at national level. All authors contributed to the article and approved the submitted version.

## Funding

This research was funded by the Food and Agriculture Organization of the United Nations (FAO—Contract number: LOA-FAVIE.2019.12– TCP/VIE/3604) and the Australian Center for International Agricultural Research (ACIAR—Contract number: C001752).

## Conflict of Interest

The authors declare that the research was conducted in the absence of any commercial or financial relationships that could be construed as a potential conflict of interest.

## Publisher's Note

All claims expressed in this article are solely those of the authors and do not necessarily represent those of their affiliated organizations, or those of the publisher, the editors and the reviewers. Any product that may be evaluated in this article, or claim that may be made by its manufacturer, is not guaranteed or endorsed by the publisher.

## Footnotes

^1^ https://cgspace.cgiar.org/handle/10568/111133

^2^See https://www.wider.unu.edu/database/2012-social-accounting-matrix-viet-nam.
