# Supplementary material for: An Assessment of the Economic Impacts of the 2019 African Swine Fever Outbreaks in Vietnam
Source: Front Vet Sci. 2021 Oct 25;8:686038. doi: 10.3389/fvets.2021.686038 (PMC8573105; doi:10.3389/fvets.2021.686038)
Supplement: Supplementary file 1 [file Data_Sheet_1.DOCX]

**Focus group discussion guidelines**

**Date:** 5/6/ 2019

**Research team:** Huyen (VNUA), lead facilitator

Giang Huong (VNUA), note taker

Trung (VNUA), board writer

Thinh (ILRI), monitor and support

**Participants:** 2 members of a cooperative

2 non-cooperative farmers

1 input supplier (feed, vet drug)

2 traders

1 slaughterhouse owner

1 processor

2 retailers

**Objective:** - Study local pig value chain

- Understand the evolution of the pig value chain overtime

**Guided questions**

1. Identify direct/indirect actors involving in the pig value chain

- What actors?

- What are their characteristics? (gender, age, wealth status, products, etc.)

- Number of actors in each node…

- Location

- ASF impacts?

2. Describe governance in the value chain

- Marketing channels?

- Who sets standards/rules?

- Are transactions price/relationship/contract based?

- ASF impacts?

3. Difficulties faced by each actor (more focus on ASF)

- Common difficulties

- Difficulties caused by ASF (adverse impacts on livelihoods)

- Coping strategies (short vs long term)
