# Supplementary material for: An Assessment of the Economic Impacts of the 2019 African Swine Fever Outbreaks in Vietnam
Source: Front Vet Sci. 2021 Oct 25;8:686038. doi: 10.3389/fvets.2021.686038 (PMC8573105; doi:10.3389/fvets.2021.686038)
Supplement: Supplementary file 2 [file Data_Sheet_2.DOCX]

**Key informant interview guidelines**

**Date:** First week of June

**Time:** 2:00 – 4:00 pm

**Interviewers:** Huyen (VNUA)

Giang Huong (VNUA)

Trung (VNUA)

Thinh (ILRI) – monitor and support

**Interviewees:** 1 district vet staff

1 commune vet staff

1 head of cooperative

2 cooperative farmers

3 non-cooperative farmers (1 small, 1 medium, 1 large)

2 traders

1 slaughterhouse

1 processor

2 retailers

2 consumers

**Interview objectives:** - understand the situation of ASF in the local context

- discuss ASF compensation scheme

**Guidelines of KIIs with district/commune vet staff**

1. **Background information**

- No. of pig farms of different scales (small, medium, large) – definition of the scales. Trends over time? Is there growth/contraction in different scales?
- Dependence of farmers on pig production by scale (role of pig production in livelihood, contribution to total income). How has dependence of scale issues changed over time? Why?
- No. of pig population – trends over time? Increasing/falling? Why?
- Main markets, market shares – trends over time? Where increasing/falling, why?
- Governance of transactions – who sets standards? Sales through markets or contracts? Are sales based on relationships or more formal means? Has this changed over time? Why? Are big buyers/companies/traders helping in any way with ASF outbreaks? If so, how?

1. **ASF**

**2.1. List ways in which the ASF outbreaks affect pig business.**

- Timeframe of ASF outbreak
- Number of affected farms (small, medium, large) – who affected the most and least? Why? Which ones affected first?
- Number of pigs infected and culled by farm scales
- Changes in sale volume and prices (relative to normal prices) – has this changed since the outbreak began? If yes, by how much?
- Changes in production practices – is this the same across different villages or farm scales? Who is changing and who is not? Why?
- Estimated losses by production scales

**2.2. List ways in which the ASF outbreaks affect other farm/non-farm activities**

What are ASF impacts on household livelihoods?

- Reduction in household expenditure (for food, schooling, other social activities)
- Sales of assets
- Reallocation of labor and capital
- Reallocation of activities – shifts towards crops or off-farm labor e.g.
- Impact of ASF on hiring off-farm labor

**2.3. Discuss government’s actions on ASF**

- List of government’s actions on ASF. Costs of compliance with specific government actions? Which ones are perceived to be affordable and which reduce disease risk? Do these actions make sense for your operation? If not, what types of actions do you do instead?
- Compensation scheme
- what %
- who gets (and what is process to obtain – steps involved)
- time to receive funds from first application? Does this vary by farmer (i.e., do more “important” or larger farmers get compensation first)?
- budget
- any difficulties faced during the implementation
- farmers’ responses
- comparison with other diseases such as FMD, HPAI, etc.)
- What could government be doing better to assist?
- Short-term and long-term scenarios of pork supply and demand under the impacts of ASF
- What has change in demand been in area for pigs? What has change in demand been in area for pork?
- How has change in demand evolved since the outbreak started? Has it become better or worse? By how much (%)
- Are consumers demanding new attributes associated with pigs/pork (traceability, quality, different cuts, fresh vs. frozen, etc.)? How are these being provided? At what costs?

**Guidelines of KIIs with head of cooperative**

1. **Background information**

- No. of cooperative members (conditions to become a member), trends over time? Why?
- Average production scale of members, trends over time? Is there growth/contraction in scales?
- Dependence on pig production (role of pig production in livelihood, contribution to total income), trends over time? How has dependence changed over time? Why?
- Functions of the cooperative in terms of input and output control?
- Main markets, market shares – trends over time? Where increasing/falling, why?
- Governance of transactions – who sets standards? Sales through markets or contracts? Are sales based on relationships or more formal means? Has this changed over time? Why?
- Advantages of members compared to non-members. Are cooperative helping in any way with ASF outbreaks? If so, how?

1. **ASF**

**2.1. List ways in which the ASF outbreaks affect pig business.**

- Timeframe of ASF outbreak
- Number of affected farms (small, medium, large) – who affected the most? Why? Which ones affected first?
- Number of pigs infected and culled by farm scales
- Changes in sale volume and prices (relative to normal prices) – has this changed since the outbreak began? If yes, by how much?
- Changes in production practices – is this the same across different villages or farm scales? Who is changing and who is not? Why?
- Estimated losses by production scales

**2.2. List ways in which the ASF outbreaks affect other farm/non-farm activities**

What are ASF impacts on household livelihoods?

- Reduction in household expenditure (for food, schooling, other social activities)
- Sales of assets
- Reallocation of labor and capital
- Reallocation of activities – shifts towards crops or off-farm labor e.g.
- Impact of ASF on hiring off-farm labor

**2.3. Discuss government’s actions on ASF**

- List of government’s actions on ASF. Costs of compliance with specific government actions? Which ones are perceived to be affordable and which reduce disease risk? Do these actions make sense for your operation? If not, what types of actions do you do instead?
- Compensation scheme
- what %
- who gets (and what is process to obtain – steps involved)
- time to receive funds from first application? Does this vary by farmer (i.e., do more “important” or larger farmers get compensation first)?
- budget
- any difficulties faced during the implementation
- farmers’ responses
- comparison with other diseases such as FMD, HPAI, etc.)
- What could government be doing better to assist?
- Short-term and long-term scenarios of pork supply and demand under the impacts of ASF
- What has change in demand been in area for pigs? What has change in demand been in area for pork?
- How has change in demand evolved since the outbreak started? Has it become better or worse? By how much (%)
- Are consumers demanding new attributes associated with pigs/pork (traceability, quality, different cuts, fresh vs. frozen, etc.)? How are these being provided? At what costs?

**Guidelines of KIIs with farmers**

1. **Background information**

- Production system (farrow to wean, wean to finish, farrow to finish) – how changed over time? Has one system become more/less important? Why?
- Number of pigs – trends over time? Increase/decrease? Why changed?
- Dependence on pig production (role of pig production in livelihood, contribution to total income) – how changed over time? Increased/decreased? Why?
- Production practices – how changed over time? Increase/decrease? Why?
- What types of biosecurity actions are they taking? What % of the costs do they represent? What constrains them from doing certain things?
- Governance of transactions – who sets standards? Sales through markets or contracts? Are sales based on relationships or more formal means? Has this changed over time? Why? Are big buyers/companies/traders helping in any way with ASF outbreaks? If so, how?

1. **ASF**

**2.1. List ways in which the ASF outbreaks affect pig business.**

- Timeframe of ASF outbreak
- Number of pigs infected and culled
- Changes in sale volume and prices (relative to normal prices) – has this changed since the outbreak began? If yes, by how much?
- Changes in production practices
- Estimated losses

**2.2. List ways in which the ASF outbreaks affect other farm/non-farm activities**

What are ASF impacts on household livelihoods?

- Reduction in household expenditure (for food, schooling, other social activities)
- Sales of assets
- Reallocation of labor and capital
- Reallocation of activities – shifts towards crops or off-farm labor e.g.
- Impact of ASF on hiring off-farm labor

**2.3. Discuss government’s actions on ASF**

- List of government’s actions on ASF. Costs of compliance with specific government actions? Which ones are perceived to be affordable and which reduce disease risk? Do these actions make sense for your operation? If not, what types of actions do you do instead?
- Compensation scheme
- what %
- who gets (and what is process to obtain – steps involved)?
- time to receive funds from first application? Does this vary by farmer (i.e., do more “important” or larger farmers get compensation first)?
- budget
- any difficulties faced during the implementation
- farmers’ responses
- comparison with other diseases such as FMD, HPAI, etc.)
- What could government be doing better to assist?
- Short-term and long-term scenarios of pork supply and demand under the impacts of ASF
- What has change in demand been in area for pigs? What has change in demand been in area for pork?
- How has change in demand evolved since the outbreak started? Has it become better or worse? By how much (%)
- Are consumers demanding new attributes associated with pigs/pork (traceability, quality, different cuts, fresh vs. frozen, etc.)? How are these being provided? At what costs?

**Guidelines of KIIs with pig traders/ processors/ retailers**

1. **Background information**

Describe how pig traders are organised and their relationships with other groups of stakeholders and trends over time

- Location of business? who are main suppliers and buyers (number)? how changed over time?
- Business scale (number of pigs traded per day/month), how changed over time?
- Governance of transactions with suppliers and buyers (who sets standards? Sales through markets or contracts? Are sales based on relationships or more formal means? Has this changed over time? Why? Any help to farmers in any way with ASF outbreaks? If so, how?
- What types of biosecurity actions are they taking? How changed over time? What % of the costs do they represent? What constrains them from doing certain things?

1. **ASF**

**2.1. List ways in which the ASF outbreaks affect pig business.**

- Timeframe of ASF outbreak
- Changes in sale volume and prices (relative to normal prices) – has this changed since the outbreak began? If yes, by how much?
- Changes in practices
- Estimated losses

**2.2. List ways in which the ASF outbreaks affect other farm/non-farm activities**

What are ASF impacts on household livelihoods?

- Reduction in household expenditure (for food, schooling, other social activities)
- Sales of assets
- Reallocation of labor and capital
- Reallocation of activities – shifts towards crops or off-farm labor e.g.
- Impact of ASF on hiring off-farm labor

**2.3. Discuss government’s actions on ASF**

- Describe actions *by the government* on ASF that affect your business (e.g. movement control during outbreaks, official and unofficial fees, roadblocks, taxes, movement permits, health certificates, inspection procedures, etc). What could government be doing better to assist?
- Describe actions by *actors in the chain* that affect your business (e.g. insistence on large volumes, quality requirements for pig, price discounts for lower quality, exclusion from certain markets, delays in payment, etc.)
- Short-term and long-term scenarios of pork supply and demand under the impacts of ASF
- What has change in demand in area for pigs? What has change in demand in area for pork?
- How has change in demand evolved since the outbreak started? Has it become better or worse? By how much (%)
- Are consumers demanding new attributes associated with pigs/pork (traceability, quality, different cuts, fresh vs. frozen, etc.)? How are these being provided? At what costs?
