# Supplementary material for: An Assessment of the Economic Impacts of the 2019 African Swine Fever Outbreaks in Vietnam
Source: Front Vet Sci. 2021 Oct 25;8:686038. doi: 10.3389/fvets.2021.686038 (PMC8573105; doi:10.3389/fvets.2021.686038)
Supplement: Supplementary file 3 [file Data_Sheet_3.docx]

**A case study of ASF impacts along the pig value chain - Specific details**

### 1. Overview of pig value chain

Animal feed suppliers

Vet drug retailers

Producers

Local slaughter-houses (Processors cum. Retailers)

Brokers

Local retailers

Local consumers

Traders

Other provinces

10%

90%

100%

50%

50%

100%

**Figure: Pig value chain in Duc Thang commune**

Discussions with FGD and KIIs revealed that the pig value chain in Duc Thang commune involved six types of actors, including input suppliers, producers, brokers, slaughterhouses, traders, and retailers. Linkages between different nodes within the value chain were generally weak and lack of coordination mechanisms existed with regard to quality, specifications, or other formal trading mechanisms. Transactions were mainly made based on informal agreements and market prices at the selling time.

Generally, pig producers sold their pigs directly to brokers or commune slaughterhouses. The FGD revealed that about 90% of the pig population was sold to brokers who had connections with traders to transport pigs to other provinces. Only 10% of pigs were consumed locally through the network of slaughterhouses. The slaughterhouses sold half of the pork to retailers operating in the district and sold the other half directly to end consumers for higher profit margins. Interactions among value chain actors were mostly based on personal relationships. Phones were the main mode of communication by which the volume and price of live pigs or pork were exchanged. Payment could be delayed or paid immediately based on the negotiation.

The qualitative assessment revealed that ASF outbreaks caused impacts on the operation patterns and livelihood of those actors in different ways. We discuss these effects on the surveyed value chain actors in turn.

### 2. Impacts of ASF on pig value chain actors

**2.1. Pig producers**

According to the FGD results, pig producers in Duc Thang commune could be classified into three categories, including small scale farmers with less than 50 pigs of all types, medium scale farmers with a total pig population ranging from 50 to 199 pigs, and large-scale farmers with more than 200 pigs. This classification was driven from respondent perspectives given the fact that there was no official classification of pig production scales in Vietnam and therefore the production scales in the literature exhibited considerable variability depending on the local context and study purposes.

Before ASF occurred in March 2019, the interviewed commune estimated it had 250 pig households with approximately 7,100 pigs, of which about 15% of its households were small scale, 75% medium scale, and 10% large scale. Due to ASF, up to the study time of 10 June 2019, around 70% of the commune’s pig herd died or culled. Nearly 30% of all pig households dropped out of business (mainly small and medium farms) as a result of ASF.

Pig production played an important role in the livelihoods of pig producers in the interviewed commune. The larger the pig production scale, the more dependent the livelihoods of pig producers. As a rough estimation from the FGD participants, approximately 20- 30% of the total household income of these small producers was derived from pig production while the figures were 50-60% and 95-100% in the case of medium and large producers, respectively. Consequently, ASF impacts on pig production and on the livelihoods of medium and large-scale ones tended to be more serious in comparison with small scale farmers. The impacts could be illustrated directly through the culling of sick pigs (applied for infected farms) or indirectly through additional expenses on biosecurity measures and the drop of market prices (applied for all farms). Before ASF, pigs were sold at around US$2.04/kg in the spring of 2019. The price then decreased sharply to US$1.61/kg right after the first ASF outbreak and then to US$1.39/kg at the time of the study. For each pig sold at an average weight of 100kg, a pig producer could thus lose as much as US$43.50 to US$65.20 per pig from this sharp price drop. In addition, expenses on disinfection increased significantly. An interview with a large pig farm revealed that before ASF, it had cost only US$2.61/week for buying disinfectants. After ASF occurred, this cost item increased by 10 times, up to US$26.10/week. Also, a reduction in household expenditure was reported by interviewed farmers of all scales. Almost all large farms obtained bank loans to invest in pig production, and thus many of them were in debt when their farms were affected by ASF. One large farm in the commune even had to sell all of their assets to cover the debts. Restocking to full capacity was not encouraged by the local authorities at the study time as the ASF virus continued to spread; thus, switching to crop cultivation, other animal species, or off-farm employment were short-term alternative strategies adopted by the affected farms regardless of scale.

**2.2. Slaughterhouses, processors, and retailers**

In the studied site, pig slaughterhouses performed multiple functions including slaughtering pigs, processing and/or retailing pork. Information from two slaughterhouses, a small and large one, were gathered from KIIs. Their main business areas were inside the residential commune and neighboring communes. The small-scale slaughterhouse interviewed reported operating at an average capacity of one to three pigs and 10-16 kg of processed pork per day. By contrast, the larger slaughterhouse had a capacity of 10 pigs and 30-40 kg of processed pork per day. After ASF, throughput was reported by KIIs to have fallen by half in all slaughterhouses regardless of scale, resulting in decreased income. However, no changes in the number of day laborers were reported among the interviewed small-scale slaughterhouses given that this business was mainly done by family members (usually a husband and wife who worked together). For the interviewed large slaughterhouse, the number of laborers was reduced from five in normal times to two after ASF.

Before ASF, the two surveyed slaughterhouses had mainly sourced pigs from small-scale and medium-scale farms (ranging from 70 to 90%), and only 10-30% of pigs from large farms.^[[1]](#footnote-1)^ After the first ASF outbreak, the volumes sourced from large farms in the commune increased to 60% and was forecasted to reach 90% in the next couple of months when small and medium farms ran out of slaughter pigs. Before ASF, the main customers of small slaughterhouses were retailers (50%) and consumers (50%) in the commune. By contrast, the largest slaughterhouse had an opportunity to supply pork to canteens of industrial zones and schools (70%) and retail channels in the city (30%). After ASF, the number of retailers sourcing pork and pork products from small-scale slaughterhouses reduced substantially. The two interviewed slaughterhouse participants noted that some small-scale slaughterhouses even stopped selling to this marketing channel altogether and completely switched to retailing pork directly to consumers. The volume supplied to canteens of the interviewed large slaughterhouse in the commune was halved.

Reducing household expenditures was mentioned by both interviewees as a strategy to cope with the income drop in the short term. In the long-term, the two surveyed respondents expressed strong beliefs that the pig sector will recover rapidly after ASF is completely eradicated, and that their business will bounce back accordingly.

**2.3. Local retailers**

There were two pork retailers in the commune. One interviewed retailer reported that they sourced pork from small slaughterhouses in the commune and sold in the temporary markets. Due to ASF, their daily trading volume reduced by half, from 20-25 kg on usual market days to 11-12kg. Their income from pork retailing decreased considerably leading to decreased household income. Retailing other meat products (e.g. chicken, beef) was considered as an alternative strategy to deal with the decrease of income from the pork business.

**2.4. Brokers**

Surveyed brokers included middlemen who helped connect pig producers and buyers and in return they would receive a commission for each successful transaction (approximately US$0.65-0.87/pig depending on price of quality of the pigs). Each commune had around one or two pig brokers. The trading volume of pig brokers changed over different periods of ASF outbreaks. Before ASF, for instance, the average volume traded per month of an interviewed broker was about 3,000 pigs. During the two first months of ASF, their sales volumes increased remarkably to 4,000 to 5,000 pigs per month because farmers proceeded with emergency sales of pigs to avoid potential risks of ASF detection on their farms. From the third month of the outbreak, the number of pigs they traded decreased to around 300 pigs because of decreasing demand and scarcity of supply, which led to a decrease of 80-89% of income from this business for these interviewed brokers.

Brokers were more likely to have income from various sources so ASF did not have much impact on their livelihoods. For instance, one interviewed broker owned one animal feed agent and one pig and poultry farm, neither of which was affected by ASF.

**2.5. Traders**

Traders participating in the study collected pigs from Northern provinces such as Hung Yen, Bac Ninh, Bac Giang, Hanoi, Hai Duong, etc. through the network of brokers to sell to large slaughterhouses in other provinces such as Hai Phong (around seven to eight slaughterhouses). A key informant interview with one trader revealed that before ASF, this trader sourced pigs from both small and medium farms (50%) and large farms (50%) with average trading volumes of 500 tons per month. After ASF, their traded volume decreased by 80% to 100 tons per month and 90 to 95% of pigs were sourced from large farms. Key informant interviews with three district and commune representatives revealed that some traders even took advantage of ASF fears to make profits by offering low prices to pig producers and then sell pigs at considerably higher market prices.

**2.6. Consumers**

After ASF was officially announced in the commune, a 50% reduction of pork demand among local consumers in the first month was reported by each of the two respondents. Demand modestly rebounded after the second month, with both estimating a 20-30% reduction compared to the period before ASF. Consumers became more cautious when choosing pork, asking carefully for the origin of pork consumed, and opting for products with traceable origins and sold by trusted distribution channels. For instance, the interview with the largest slaughterhouse in the commune that opened a pork retail shop revealed that its sales volume has increased by 20% since ASF. In addition, local consumers were likely to switch from buying pork in the market to purchasing live pigs from reliable sources to self-slaughter and store in refrigerators for gradual consumption.

**2.7. Animal feed and vet drug suppliers**

There were seven animal feed suppliers and three vet drug suppliers in the commune. Their main customers were small-scale and medium-scale farms because most large farms sourced animal feed and vet drugs directly from companies. Before ASF, one interviewed feed supplier stated that they could sell an average of 150 tons of animal feed per month. Since the outbreak of ASF, over the past three months, this supplier only managed to sell 20 tons per month and the number of regular customers also dropped by 15%. This feed supplier further revealed that their greatest difficulty with input suppliers during ASF was to collect debt from pig producers. Usually, animal feed suppliers had to pay the manufacturers on receipt of the feed while often selling feed to farmers on credits. Due to ASF, pigs were infected and culled so farmers did not have money to pay back to suppliers.

### 3. Impacts of ASF on governance and coordination mechanisms between actors

Results from the FGD and KIIs with various actors revealed significant changes in the governance of transactions along the pig chain in the commune due to ASF. For instance, before ASF, surveyed pig producers could buy animal feed on credit; however, after ASF, this payment method was not accepted by any of the feed suppliers. In addition, pig buyers used to enter pig pens freely to see the pigs before deciding whether to buy or not, and payment had been made in cash on the spot. After ASF, pigs were shown to buyers through camera or apps (Zalo, Viber, etc.) rather than direct observation. After reaching an agreement on quantity and price, the farms weighed and loaded pigs to trucks that must park far outside the farms. Payment was either in cash or transferred through bank accounts. One interviewed large-scale farmer even reported that *“I put cash into hot water after receiving from the buyers to avoid the possibility of ASF virus transmission.”*

Local slaughterhouses also became more selective in selecting pigs for slaughtering as a strategy to win customer trust and keep their reputation in the context of rising food safety concerns during ASF. Given that local slaughterhouses operated their business in residential areas where everyone knew each other, a bad case reported by a consumer could completely ruin the whole business. Thus, one interviewed slaughterhouse was willing to pay more (they estimated from 7 to 10% higher than the normal market price) to purchase high quality pigs. By contrast, brokers and traders were more likely to loosen their selection standards. Interviews with one broker and one trader revealed that during ASF they bought all types of pigs regardless of quality and health status. Healthy pigs sold at market prices while sick pigs received a fixed disposal price of US$43.5/head. Consequently, pig producers preferred selling pigs to traders rather than to local slaughterhouses, especially for those farms which had pigs suspected of being infected by ASF.

Since ASF occurred, an example of greater coordination within the value chain was observed with the establishment of a farmer cooperative named Duc Thang cooperative. An interview with a representative of the cooperative revealed that even though the cooperative included only 18 out of 250 pigs farmers in the commune, its pig herd accounted for 46% of total pig population of the whole commune. Participating farmers must meet requirements such as a common interest of pig raising, applying Vietnamese good animal husbandry practices (VietGAHP) in production, locating pig farms outside the residential areas, having 20-30 sows and 60-80 fattened pigs, and contributing US$869 to the charter capital of the cooperative. The cooperative helped link their members with input suppliers and market outlets via contractual arrangements. It signed contracts with the Charoen Pokphand (CP) feed company and a vet drug company so their members could get high quality inputs with better price offers and accompanying technical support. For instance, the vet drug company regularly sent their staff to the farms of cooperative members to help with vaccination. The cooperative also arranged contracts with two of the largest slaughterhouses in the district to supply approximately 500 pigs per month. To deal with ASF, the cooperative allocated funds to buy disinfectants and lime for members to increase disinfection around farms. Meetings were organized more regularly for cooperative farm members to update on the ASF situation, introduce effective preventive and control measures, and facilitate the supply of breeding pigs (piglets produced by members should be prioritized to supply to other members in need before selling outside). The cooperative also proactively contacted pig traders from other provinces to purchase pigs from its members when the contracted slaughterhouses reduced capacity. Up to the date of the study, only two farms of the cooperative had ASF.

1. These ranges are derived from one slaughterhouse reporting it sourced 85-90% of pigs from small-scale and medium farms, with the remaining 10-15% from large-scale farms, while the other interviewed slaughterhouse noted sourcing 70-90% of pigs from small-scale and medium sized farms and the remainder from large-scale farms. [↑](#footnote-ref-1)
